# Exploring the link between cognitive deficit, self‐esteem, alexithymia, and depressive symptom of schizophrenia

**DOI:** 10.1002/brb3.2648

**Published:** 2022-06-08

**Authors:** Chen He, Xueying Zhang, Qingrong Xia, Hua Gao, Junwei Yan, Xuequan Chen, Hui Yuan, Yang Zhang, Wen Xie, Cuizhen Zhu

**Affiliations:** ^1^ Affiliated Psychological Hospital of Anhui Medical University Hefei Anhui China; ^2^ Anhui Clinical Center for Mental and Psychological Diseases Hefei Fourth People's Hospital Hefei Anhui China; ^3^ Anhui Mental Health Center Hefei Anhui China

**Keywords:** alexithymia, cognitive deficit, depressive symptoms, schizophrenia, self‐esteem

## Abstract

**Objective:**

To the best of our knowledge, studies have been rarely conducted to assess the correlation between cognitive deficit, self‐esteem, and alexithymia in the depressive symptoms of schizophrenia (SCZ). Therefore, this study aims to explore the risk factors associated with impairment of cognitive function, alexithymia, and self‐esteem among a representative sample of first‐episode schizophrenic patients.

**Method:**

We recruited 107 first‐episode schizophrenic patients (48.6% male, 51.4% female, 36.94 ± 10.73 years) into the research group, according to the Diagnostic and Statistical Manual of Mental Disorders (5th edition). A total of 45 healthy people (51.1% male, 48.9% female, 32.47 ± 10.94 years) were enlisted in the healthy control group. Psychotic symptoms were evaluated using the Positive and Negative Syndrome Scale (PANSS). Cognitive functions were estimated using the Montreal Cognitive Assessment Scale (MoCA). The feelings of respect and self‐acceptance were tested using the Rosenberg Self‐Esteem Scale (RSES). Emotion of identifying and describing were measured by self‐report scale of Toronto Alexithymia Scale‐20 (TAS‐20).

**Results:**

Overall cognitive impairment and alexithymia were found more serious in the patients of SCZ than the healthy group (*p *< .001, respectively). The patients of SCZ have higher self‐esteem than the healthy group (*p = *.013). Total score of MoCA, ability of visual space and executive function, and delayed recall were explored had negatively correlation with alexithymia (*r *= −.319, *p *= .001; *r *= −.248, *p = *.010; *r = −*0.263, *p = *.006). Total score of RSES and depressive symptoms of PANSS had a positive correlation with alexithymia (*r *= .394, *p *= .001; *r *= .208, *p = *.032). Stepwise regression analyses have shown a positive relationship between difficulty describing feelings and depression subscale of PANSS (*β = *.188, *t *= −2.007, *p = *.047) while a negative relationship between externally oriented thinking and depression subscale of PANSS (*β = *−.244, *t* = −2.603, *p = *.011). A positive link correlation also was found between the total scores of TAS and RSES (*β *= .372, *t *= 4.144, *p *= .001). A negative relevance was found between the total scores of TAS and scores of MoCA (*β *= −.305, *t *= −3.348, *p *= .001).

**Conclusion:**

Overall impairment of cognitive function and alexithymia are commonly encountered in SCZ patients. Poor cognitive function, alexithymia, and high level self‐esteem may be specific detective risk factors for the depressive symptoms of SCZ.

## Graphical abstract

This study aims to explore the risk factors associated with impairment of cognitive function, alexithymia, and self‐esteem among a representative sample of first‐episode schizophrenic patients. High self‐esteem and alexithymia are commonly encountered in schizophrenia patients. Poor cognitive function, self‐esteem, and alexithymia may be a specific detective risk factor for the depressive symptoms of SCZ.

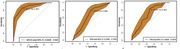

## 1 INTRODUCTION

Schizophrenia (SCZ) is a serious mental illness characterized by symptoms of positive, negative, cognitive, affective, and reality testing domains (Valle, 2020). It is a leading cause for disability and affects approximately 1% the population worldwide (Kraguljac et al., 2013). Globally, epidemiological studies have demonstrated that prevalent cases of patients with SCZ rose from 13.1 million in 1990 to 20.9 million in 2016 (Charlson et al., 2018). Presently, second‐generation antipsychotic drugs (SGAs), which are effective in relieving positive symptoms of the disease and have a low propensity to cause extrapyramidal side effects, became the mainstay of SCZ treatment (Divac et al., 2014). However, in view of the lack of efficacy improvement regarding negative symptoms, metabolic syndrome, and cognitive function, the SGAs were limited in clinical application (Divac et al., 2014). Thus, scientists are looking for the methods of nonpsychotropic drugs to effectively improve these side effects, namely, calligraphy activity (W. Y. Huang et al., 2022), psychotherapy interventions (Roberts et al., 2021), aerobic exercise (Firth et al., 2017), and so forth. Early clinical research reported that the presence of moderate to severe depressive symptoms can occur at any point during SCZ (J. Huang et al., 2019). The results of prevalence rate have shown that approximately 30−70% of patients with SCZ suffer from depressive symptoms, which could be directly linked to poor functional outcomes, poor treatment outcomes, high morbidity, and suicidal behavior (Fang et al., 2019). In this context, close inspection and examination are important to address the depressive symptoms in SCZ and deepen our understanding of its pathophysiology.

Numerous studies have found that depressive and cognitive symptoms in SCZ share many similar characteristics (Sheffield et al., 2018). In particular, they have been reported to be linked to the course, prognostic importance, and various aspects of everyday functional skills performance of patients with SCZ (Upthegrove et al., 2017; Yang et al., 2018). Structural brain imaging studies have explored indirect support that the depressive symptoms in SCZ (Kohler et al., 1998), specifically, ventromedial prefrontal cortex, played a multifaceted role in neuropsychological impairment, namely, abstraction, mental flexibility, attention/vigilance, verbal memory (Liu et al., 2021), learning (Kohler et al., 1998), emotion, decision‐making, and social cognition (Hiser & Koenigs, 2018). These findings show that the depressive symptoms and cognition function in SCZ involves similar brain systems regardless of its etiology. Impairment of cognitive symptoms might stem from perturbation of various performance tasks, such as processing speed (Rodriguez‐Sanchez et al., 2007), memory performance (Roofeh et al., 2006), attention deficit (A. Katsumi et al., 2019), reasoning difficulty, and problem solving (Firth et al., 2017). Neuropsychological tests have demonstrated that severe global cognitive impairment in adults with SCZ would be a particularly important risk predictor from the perspective of treatment (Yolland et al., 2021). In recent years, the number of cognitive remediation interventions have improved functionally in SCZ patients with respect to emotional regulation, processing speed, attention/vigilance, working memory, verbal learning and fluency, and executive function (A. Katsumi et al., 2019). However, a consensus has not been reached concerning what impairment global cognitive function and depressed mood are effectively improved by cognitive remediation (Katsumi et al., 2015). So far, these heterogeneous outcomes are also a reminder that a great deal of unknown distinct mechanisms in the global cognitive deficits and mood have not been clarified, which could be a potential avenue for the treatment of psychotic symptoms.

Alexithymia is broadly characterized as a range of interrelated difficulties to identify feelings and distinguish between feelings and bodily sensations of emotional arousal as well as a deficit in the ability to consciously experience, identify, and express emotions (Fogley et al., 2014), and focuses a specific impediment on awareness and expression of one's own emotions (Tang et al., 2016). Several authors have reported that alexithymia may be a vulnerability factor in the development of SCZ, but knowledge concerning this underpinning of deficits in emotion self‐awareness in SCZ remains unclear. Previous studies have found that schizophrenic patients have unusual symptoms of affective disturbance (Herbener et al., 2005), whereby they experience emotion less frequently and intensely than healthy individuals (Horan & Blanchard, 2003). Furthermore, a range of studies has sought to investigate the presence of alexithymia among patients with SCZ by examining its links to features of symptoms (van ’t Wout et al., 2007). Research has demonstrated that the presence of alexithymia in SCZ may result in persistent suicidal thoughts, more severe depressive symptoms, and sleep problems independently by the severity of positive and negative symptoms (Alimoradi et al., 2022; Demirkol et al., 2019). In particular, researchers found that these features of alexithymia persist during periods of clinical stability rather than appearing secondary to psychotic symptoms (Wingbermuhle et al., 2012). Until the present, few studies have examined cognition impairment, depressive symptoms, and alexithymia simultaneously in SCZ. An improved understanding of the links between depressive symptoms, cognitive deficits, and alexithymia seems essential to better comprehend the functional impairments and poor therapeutic outcomes of SCZ.

To clarify this issue, we hypothesized that alexithymia could be related to the depressive symptoms and recognition deficits in SCZ. Further effort is needed to address the possible link between alexithymia and depressive symptoms and in specific deficits of cognition. Our primary prediction was that greater levels of alexithymia would be related to depressive symptoms in SCZ, and a range of different types of alexithymia are linked to poorer cognitive domains. Furthermore, we would observe how specific domain of alexithymia affects the psychotic depressive symptom and the precise pattern of cognitive deficit. Finally, we aimed to explore the predictive ability of alexithymia and related susceptible factors in our research, and we anticipate that alexithymia would account for some of the predictive variance on functional outcome in our overall sample.

## 2 MATERIAL AND METHODS

### 2.1 Participants

This cross‐sectional cohort research was conducted at Anhui Mental Health Center (AMHC) between February 2019 and September 2021.

Two senior psychiatrists used the Mini‐International Neuropsychiatric Interview (MINI) 6.0.0 to confirm the psychiatric diagnosis of all participants; in addition, two trained psychiatric residents independently scored for each participants with the related scales, the mean scores taken by them were used as the final score.

Patients in the episode group met the following inclusion criteria: (1) fulfillment of the fifth edition of the Diagnostic and Statistical Manual of Mental Disorders (DSM‐5) criteria for first episode patients with SCZ by two independent experienced psychiatrists, (2) aged between 16 and 65 years old, (3) and with no history of traumatic brain injury or any neurological disease.

A total of 235 inpatients with first episode of SCZ were initially evaluated. Among which 22 individuals screened failures, 20 refused to participate in this study, and 86 who did not meet the experimental criteria were excluded from this study.

Hence, 107 inpatients with first episode of SCZ were included into the case group (SCZ group = 107).

Individuals in the healthy group met the following inclusion criteria: (1) aged between 16 and 65 years old, (2) healthy volunteers were assessed with MINI 6.0.0 to rule out any psychiatric conditions. All the participants did not have any of the following exclusion criteria: (1) history of craniocerebral traumatic injury or neurological disease or severe somatic diseases, (2) history of alcohol or other substance abuse, (3) pregnant or lactating women, (4) and history of convulsive electroconvulsive therapy within three months.

Finally, 45 healthy people were recruited from the physical examination center of AMHC into the normal control group (healthy group, *n *= 45).

**FIGURE 1:**
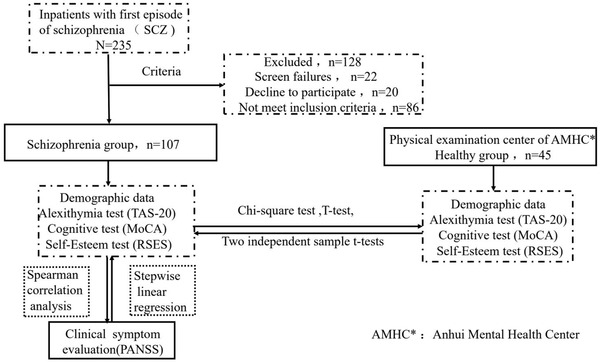
Flow chart

Ultimately, a total of 152 participants were included into the different two groups in this study (Figure 1). Distribution of mean age was similar in the two groups, namely, 36.94 (SD = 10.73) for individuals with SCZ and 32.47 (SD = 10.94) for controls (*p *= .356).

The study was approved by the Medical Ethics Committee of AMHC. All individuals provided written consent prior to research participation in accordance with the principles of the Declaration of Helsinki. The trial clinical registration number was ChiCTR2100045240.

### 2.2 Assessments MINI 6.0.0

MINI 6.0.0, which is a brief and straightforward diagnostic interview for psychiatric disorders used by psychiatrists in the United States and Europe, was used to confirm the preliminary clinical diagnosis. The reliability inter‐rater and test–retest of MINI 6.0.0 were excellent and its kappa values were above .80 and .90, respectively. All patients underwent MINI to verify the clinical diagnoses of first‐episode SCZ patients (Kadri et al., 2005).

### 2.3 Positive and negative syndrome scale

The Positive and negative syndrome scale (PANSS) is the gold standard for assessing severe psychotic symptoms through a semistructured interview. The PANSS is a 30‐item rating scale used to assess the dimensions of SCZ symptoms and the severity of symptoms in the present study. Principal component analysis revealed a six‐factor structure for the patients of the first episode group: (1) insufficiency of response, (2) thinking disturbance, (3) activity, (4) paranoid, (5) depression, and (6) aggressiveness (Dragioti et al., 2017).

### 2.4 Toronto alexithymia scale 20

The Toronto alexithymia scale 20 (TAS‐20) (TAS‐20) is composed of 20 self‐report items used to evaluate the affective and cognitive aspects of alexithymia. It contains three factor sub scores: difficulty identifying feelings, difficulty describing feelings, and externally oriented thinking. The range of the TAS‐20 total score is 20−100, with higher scores indicating greater severity of alexithymia. The TAS‐20 has demonstrated good internal consistency and retest reliability (Bagby et al., 1994).

### 2.5 Montreal cognitive assessment scale

Montreal cognitive assessment scale (MoCA) consists of a total of 30 items used to examine short‐term memory, attention and working memory, and executive functions, which are commonly cognitive impairment in patients with SCZ. Scores of MoCA range from 0 to 30 points. If the score is lower than 25, it can be regarded as cognitive impairment, then this cutoff is now widely used as a threshold for detecting cognitive impairment and possible dementia. The internal consistency of MoCA was verified to be good, with the Cronbach's alpha of the standardized program being .83 (Rosca et al., 2020).

### 2.6 Rosenberg self‐esteem scale

Rosenberg self‐esteem scale (RSES) is one of the most widely used methods for assessing global self‐esteem; it consist 10 items that are mainly centered on the feelings of respect and self‐acceptance. Half of the items are scored positively and the other half negatively. The range of scale is from 1 = agree to 4 = totally disagree. It has a good reliability of .74 and validity of .87; the internal consistency reliability of SES was verified to be good, with Cronbach's alpha of the standardized program being .81 (Sinclair et al., 2010).

### 2.7 Statistical analysis

The counting and variable data of demographic characteristic differences between two groups were compared by chi‐square test and T‐test, respectively. Two independent sample t‐tests were used to compare the scores of TAS‐20, MoCA, and RSES between the first episode and control groups. Spearman correlation analysis was used to test the relationship between possible correlation factors and alexithymia scale scores. Stepwise linear regression analysis was used to explore the relationship and interaction between risk factors, psychiatric symptoms, cognitive function, self‐stigma, and alexithymia in patients in the first‐episode group. The area under the receiver‐operating characteristic curve was used to investigate the impairment of cognitive function, alexithymia, and self‐stigma to evaluate the severity of psychotic symptoms of SCZ patients.

The area under the receiver‐operating characteristic curve (ROC curve) was used to assess the metrics of sensitivity and specificity at the optimal cutoff value of 0.5. In the present study, chi‐square test, T‐test, and Spearman correlation analysis were conducted using the SPSS version 22.0 (IBM Corp). ROC curve analysis was performed using OmicStudio tools at https://www.omicstudio.cn/tool/58. All statistical tests were two‐tailed, and statistical significance was set as *α *< .05.

## 3 RESULTS

### 3.1 Characteristic results of demographics, cognition function, alexithymia, and feelings of self‐esteem between two groups

Sociodemographic and clinical characteristics of participants classified into categories are shown in Tables 1 and  2. The final sample was similar included 48.6% (*n* = 52) males and 51.4% (*n* = 55) females in the SCZ group versus 51.1% (n = 23) males and 48.9% (*n* = 22) females in the healthy group (*χ* ^2^ = 0.080, *p *= .777). Regarding years of education, marital status, and occupation, the two groups also had no significant difference (*χ* ^2^ = 3.749, *p *= .053; *χ* ^2^ = 3.538, *p *= .060; *χ* ^2^ = 3.578, *p *= .059, respectively). We investigated the characteristics of alexithymia, cognition function, and feelings of esteem between the two groups. The results showed that the overall cognitive impairment and alexithymia were be found more serious in the patients of SCZ than the healthy group (*p *< .001, respectively). The patients of SCZ have higher self‐esteem than the healthy group (*p = *.013). Furthermore, we found the three dimension scores of alexithymia subscales in schizophrenic patients were higher than those in the normal control group, including difficulty identifying feelings (*T *= 2.381, *p *= .019), difficulty describing feelings (*T *= 3.777, *p *< .001), and externally oriented thinking (*T *= 4.744, *p *< .001). Likewise, impairment of cognitive function in the patients with SCZ was more prominent than that in the healthy group, including scores of orientation, attention, reading and so forth (*p*<.001, respectively).

**TABLE 1:** Comparison of sociodemographic characteristics between two groups

Factors	Schizophrenia group (*n* = 107)	Healthy group (*n* = 45)	*t*/*χ* ^2^	*p*
Age^a^(years)	36.94 ± 10.73	32.47 ± 10.94	0.852	.356
Sex				
Male	52 (48.6%)	23 (51.1%)	0.080	.777
Female	55 (51.4%)	22 (48.9%)		
Years of education (years)				
≤9	49 (45.8%)	13 (28.9%)	3.749	.053
> 9	58 (54.2%)	32 (71.1%)		
Marital status				
No	72 (67.3%)	23 (51.1%)	3.538	.060
Yes	35 (33.7%)	22 (48.9%)		
Employment status				
No	56 (52.3%)	16 (35.6%)	3.578	.059
Yes	51 (47.7%)	29 (64.4%)		

^a^ Mean ± standard deviation.

**TABLE 2:** Comparison of cognition function, self‐esteem, and alexithymia between two groups

Scales	Schizophrenia group (*n* = 107)	Healthy group (*n* = 45)	T	*p*
Total scores of TAS	55.11 ± 10.433	48.378 ± 7.941	4.330	<.001
F1(difficulty identifying feelings)	18.41 ± 6.177	16.178 ± 4.854	2.381	.019
F2(difficulty describing feelings)	13.85 ± 3.784	11.867 ± 2.528	3.777	<.001
F3(externally‐oriented thinking)	22.85 ± 3.059	20.333 ± 2.804	4.744	<.001
Total scores of MOCA	19.80 ± 4.783	26.289 ± 2.944	−10.174	<.001
Orientation	5.33 ± 1.188	5.889 ± 0.318	−4.523	<.001
Naming	2.589 ± 0.777	2.844 ± 0.424	−2.605	.010
Attention	1.682 ± 0.488	1.911 ± 0.288	−3.591	<.001
Reading	0.523 ± 0.502	0.933 ± 0.252	−6.679	<.001
Language	1.692 ± 1.068	2.533 ± 0.625	−6.053	<.001
Abstraction ability	1.150 ± 0.845	1.689 ± 0.596	−4.469	<.001
Delayed recall	1.664 ± 1.704	3.556 ± 0.813	−9.249	<.001
100‐7(times)	2.533 ± 0.925	2.889 ± 0.438	−3.217	.002
Visual space and executive function	2.645 ± 1.416	4.044 ± 1.381	−5.604	<.001
Total scores of RSES	17.336 ± 3.826	15.689 ± 3.377	2.506	.013

*Note*: Data presented as Mean ± SD or *N*.

Abbreviations: MOCA, Montreal Cognitive Assessment Scale; RSES, Rosenberg Self‐Esteem Scale, TAS, Toronto Alexithymia Scale‐20.

### 3.2 Factors correlated between alexithymia with cognition function, psychotic symptoms, and self‐esteem

To evaluate the potential risk factors correlated between alexithymia with impairment cognition function, psychotic symptoms, and self‐esteem, we used Spearman correlation analysis to assess how alexithymia affects the psychotic symptom, precise dimension of cognitive deficits, and self‐esteem; the results are shown in Table 3, 4. Firstly, we found the main negative effect of three dimensions and total score of alexithymia on the total score of cognitive impairment severity (*r *= −.265, *p = *.006; *r *= −.283, *p = *.003; *r *= −.213, *p = *.028; *r *= −.319, *p *= .001, respectively), ability of visual space and executive function (*r *= −.201, *p = *.038; *r *= −.203, *p = *.036; *r *= −.199, *p = *.040; *r *= −.248, *p *= .010, respectively), and two dimensions and total score of alexithymia on the delayed recall (*r *= −.278, *p = *.004; *r *= −.262, *p = *.006; *r *= −0.263, *p = *.006, respectively). Secondly, we also discovered that the two dimensions of alexithymia (difficulty describing feelings and externally oriented thinking) and total scores of alexithymia had a positive correlation with the depressive symptoms of PANSS subscale (*r *= .207, *p = *.033; *r *= .218, *p = *.024; *r *= .208, *p = *.032, respectively). Interestingly, the results of the present study explored that self‐esteem was positively correlated with difficulty identifying feelings, difficulty describing feelings, and total scores of alexithymia (*r *= .328, *p = *.001; *r *= .347, *p = *.001; *r *= .394, *p = *.001, respectively).

**TABLE 3:** Correlation analysis between sociodemographic characteristics, cognition function, self‐esteem, and psychiatric symptoms with alexithymia

	F1		F2		F3		Total score of TAS	
Factors	*r*	*p*	*r*	*p*	*r*	*p*	*r*	*p*
Sex	.008	.931	.086	.380	.219*	.023	.088	.368
Age	.165	.090	.168	.083	.150	.122	.199*	.040
Marital status	−.005	.961	.059	.546	.175	.071	.043	.657
Employment status	−.105	.282	−.140	.150	−.180	.063	−.169	.081
Years of education	−.133	.172	−.164	.091	−.119	.221	−.175	.072
BMI	−.104	.285	−.166	.087	−.038	.694	−.106	.276
**Total score of MOCA**	−.265**	.006	−.283**	.003	−.213*	.028	−.319**	.001
Visual space and executive function	−.201*	.038	−.203*	.036	−.199*	.040	−.248*	.010
Naming	−.020	.839	.006	.950	−.102	.297	−.052	.595
Attention	−.079	.420	−.078	.427	−.208*	.031	−.150	.123
Reading	−.030	.761	−.002	.985	−.099	.312	−.041	.678
100‐7 (times)	−.044	.652	−.113	.248	−.167	.085	−.108	.270
Language	−.121	.213	−.104	.287	−.136	.164	−.140	.150
Abstraction ability	−.051	.600	−.131	.179	−.143	.141	−.121	.213
Delayed recall	−.278**	.004	−.262**	.006	−.048	.621	−.263**	.006
Orientation	−.159	.101	−.240*	.013	−.019	.849	−.195*	.044
**Total score of PANSS**	.080	.411	.063	.519	.078	.424	.083	.397
F1 (insufficiency of response)	.082	.404	.021	.827	−.039	.689	.042	.664
F2 (thinking disturbance)	.100	.304	.082	.399	−.135	.164	.046	.636
F3 (activity)	−.009	.924	.067	.496	.020	.836	.028	.773
F4 (paranoid)	.117	.229	−.022	.822	.114	.241	.104	.286
F5 (depression)	.153	.116	.207*	.033	.218*	.024	.208*	.032
F6 (aggressiveness)	.004	.971	.018	.856	.099	.310	.043	.663
Total score of RSES	.328**	.001	.347**	.001	.188	.052	.394**	.001

Abbreviations: BMI, body mass index; F1, difficulty identifying feelings; F2, difficulty describing feelings; F3, externally‐oriented thinking; MOCA, Montreal Cognitive Assessment Scale; PANSS, Positive and negative symptoms; RSES, Rosenberg Self‐Esteem Scale; TAS, Toronto Alexithymia Scale‐20.

*p ≤ .05, **p ≤. 001.

**TABLE 4:** Stepwise linear regression analysis of cognitive function)

Dependent variables	Independent variables	B	SE	Beta (*β*)	*t*	*p*	*R* ^2^
PANSS F5	TAS‐F2	.120	0.060	.188	2.007	.047	.108
	TAS‐F3	−0.192	0.074	−.244	−2.603	.011	
Total score of RSES	Total score of TAS	0.137	0.033	.372	4.144	.001	.185
Total score of MOCA	Total score of TAS	−0.140	0.042	−.305	−3.348	.001	.137

Abbreviations: B, unstandardized coefficient; Beta, standardized coefficient; F2, difficulty describing feelings; F3, externally oriented thinking; MOCA, Montreal Cognitive Assessment Scale; *R*2: R square; SE, standard error; RSES, Rosenberg Self‐Esteem Scale; TAS, Toronto Alexithymia Scale‐20.

*p ≤ .05, **p ≤ .001.

### 3.3 Independent predictors of cognition function, psychotic symptoms, and self‐esteem

Stepwise regression analyses of sociodemographic characteristics, cognition function, psychotic symptoms, and self‐esteem were performed to predict risk factors for alexithymia. The final model from forward regression indicated that the remarkable explanatory variables accounted for 10.8% of the variance in depression subscale of PANSS. The depression subscale of PANSS showed a significant positive relationship with difficulty describing feelings (*β = *.188, *t *= −2.007, *p = *.047), and negative relationship with externally oriented thinking (*β = *−.244, *t = *−2.603, *p = *.011). Furthermore, the final model from forward regression indicated that the remarkable explanatory variables accounted for 18.5% of the variance in RSES. A positive correlation was observed between the total scores of TAS and RSES (*β *= .372, *t *= 4.144, *p *= .001). Ultimately, the final model from forward regression indicated that the remarkable explanatory variables accounted for 13.7% of the variance in total scores of MoCA. The total scores of TAS had negatively relevant to scores of MoCA (*β *= −.305, *t *= 3.348, *p *= .001).

**FIGURE 2:**
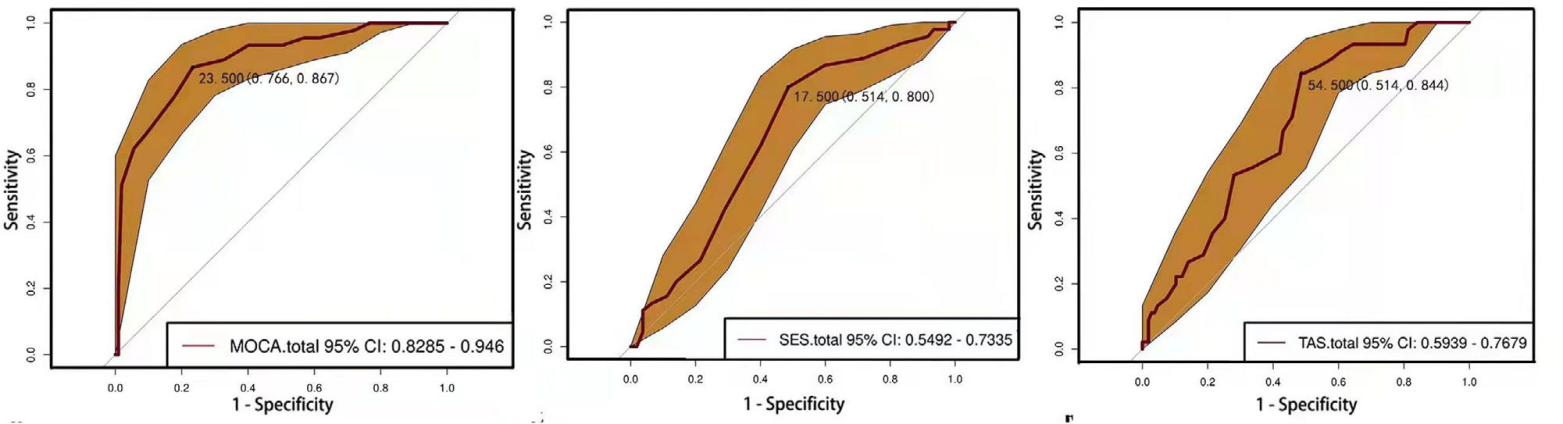
ROC curve analysis. It was used to predict the cognitive function impairment, alexithymia, and self‐esteem of SCZ group by using clinical symptoms, and 95% confidence interval

ROC curve analysis was used to predict the severity of psychotic symptoms of SCZ patients by using total score of MoCA, total score of TAS, and total score of RSES. Total score of MoCA was able to predict the severity of psychotic symptoms of SCZ patients at a cutoff level of 23.5 scores, with a specificity of 0.766 and sensitivity of 0.867 (Figure 2a). Total score of TAS can predict severity of psychotic symptoms of SCZ patients at a cutoff level of 54.5 scores, with a specificity of 0.514 and a sensitivity of 0.844 (Figure 2b). Total score of RSES can predict the severity of psychotic symptoms of SCZ patients at a cutoff level of 17.5, with a specificity of 0.514 and a sensitivity of 0.800 (Figure 2c).

## 4 DISCUSSION

The main aim of the present study was to investigate the relationship between depressive symptoms, impairment of cognitive function, and alexithymia in SCZ. The results demonstrated that patients of SCZ suffered more difficulty in identifying feelings, describing feelings, and externally oriented thinking than the normal control group. We also found that overall impairment of cognitive function in the patients with SCZ was more aggrieved than that of the healthy group. The SCZ patients represented the lower level of self‐esteem and alexithymia than the healthy group. Furthermore, we discovered that depressive symptoms and high level of RSES had a positive correlation to alexithymia, while depressive symptoms and cognitive function had a negative correlation with it. In addition, we also found that cognitive impairment and high levels of TAS and RSES could have a high specificity and be a sensitivity risk predictor for the severity of depressive symptoms of SCZ patients.

In line with previous studies, our work observed that the cognitive impairment of SCZ patients was more severe than that of healthy people (Sheffield et al., 2018). In general, SCZ is considered a neurodevelopmental disorder, and the interaction context of genetic and environmental risk factors may contribute to its etiology (Rapoport et al., 2012). There is no mystery regarding whether cognitive impairment is in SCZ (Mesholam‐Gately et al., 2009), and cognitive deficits are a core feature of SCZ that provide a window to a richer understanding of the occurrence, transversion, and prognosis of diseases. Many longitudinal studies have exposed cognitive deficits as a robust predictor and contribute to functional capacity impairment in daily life (Green et al., 2004). Thus, examining the unique type of cognitive impairment in SCZ allows for assessment of differential trajectories. Previous scholars found that relatively stable cognitive deficits were observed in SCZ, including attention/vigilance, working memory, verbal fluency, visual learning, and processing speed abilities (Sheffield et al., 2014). These cognitive impairments may contribute to work functional disability and social functional disability in the real independent living world across the course of illness (Kern et al., 2011), especially in interpersonal skills, work skills, and community activities (Gaweda & Krezolek, 2019). Similar to those findings, the present study explored that the poor ability of visual spatial function, executive function, and delayed recall were related to the difficulties in identification of feelings, describing feelings, and externally oriented thinking, which predicts more impaired real‐world functional capacity in SCZ (Gaweda & Krezolek, 2019). It will become increasingly important to deepen our understanding of how dysfunction changes in the relationship between cognitive impairment and alexithymia among schizophrenic patients.

Our results indicated that difficulty describing feelings, externally oriented thinking, and high severity of alexithymia were positively related to the depressive symptoms of PANSS; this finding is consistent with that of many previous studies (Faramarzi & Khafri, 2017). Alexithymia is considered to be a kind of ability to understand, process, and describe feelings. Researchers also recommended that individuals high in alexithymia have difficulties in identifying feelings and emotion regulation (Vanheule, 2008), which may be a disadvantage in managing stressors and emotion‐triggering events. Depressed people who have difficulty in identifying and describing feelings may have a limited capacity to communicate their gloomy feelings to others, which in turn causes the depressed people are less capable of cognitive reappraisal and an effective coping strategy (Saarijarvi et al., 2006). Nevertheless, previous studies have mostly focused on depression, anxiety, chronic benign pain, obesity, and somatic diseases. Limited attention has been paid to investigate the linked correlation between alexithymia and depressive symptoms of SCZ. In the long term, this impairment of ability may hinder and delay the intervention for mood disorders.

More recently, evidence has supported the notion that implicit self‐esteem is an independent predictive mediating risk factor for depression onset and depression recurrence, showing that it could be a relevant target for intervention (David et al., 2008). Low self‐esteem is subjective with a low general assessment of the self, feelings of worthlessness, and self‐doubt. In most states, depressive symptoms, low self‐esteem, and alexithymia are co‐occurring (Faramarzi & Khafri, 2017). Thus, to better understand the role of self‐esteem in the correlation between alexithymia and depressive symptoms of PANSS, we performed Spearman's correlation analysis and stepwise linear regression analysis, which showed that self‐esteem level had a positive relationship with difficulty in identifying and describing feelings, and externally oriented thinking. In addition, we also found that alexithymia, impairment of cognitive function, and self‐esteem level had high predictive validity of sensitivity and effectiveness for depressive symptoms of SCZ. Considering all the aforementioned results, poor cognitive function, alexithymia, and self‐esteem level showed overlapped prognostic value for the development of depressive symptoms of SCZ that could be independent risk factors to predictive qualities simultaneously observed in schizophrenic patients.

Several limitations of the current findings should be considered in this context. First, the main limitation is relatively small sample sizes in the present study and is needed to increase sample sizes to detect statistical power. Second, this study is cross‐sectional research, and a long‐term longitudinal research may be conducted to clarify the causal relationship between impaired cognitive function, alexithymia, low self‐esteem, and depressive symptoms of SCZ. Finally, and importantly, it is reported that some important confounders, such as self‐stigma, game disorder, coping avoidance, and sleep quality (K. C. Chang et al., 2022; Y. H. Chang et al., 2020), are related to psychological distress; these factors should therefore be controlled in the future study. The current study also has several strengths. First is the use of more homogeneous first‐episode SCZ patients. In this way, we could minimize the potential confounding factors of antipsychotic drugs. Second, few studies pay close attention to relationship between low self‐esteem, impaired cognitive function, and alexithymia in patients with depressive symptoms of SCZ. These results may supply evidence in terms of difficulties in describing emotions, and low self‐esteem may cause schizophrenic patients to realize that they have difficulties with social support, which may increase the impact on the severity of depressive symptoms of SCZ.

In conclusion, impaired cognitive function, self‐esteem level, and alexithymia are commonly encountered in SCZ patients. Overall, both poor cognitive function and self‐esteem level had varying degrees of impact on alexithymia. Impaired cognitive function, self‐esteem level, and alexithymia may be a specific detective risk factor for the depressive symptoms of SCZ. A comprehensive understanding in this realm will likely be beneficial to schizophrenic patients in future treatment and intervention.

## CONFLICT OF INTEREST

None declared.

## AUTHOR CONTRIBUTIONS

Cuizhen Zhu and Wen Xie were responsible for study design and manuscript editing. Chen He and Xueying Zhang were responsible for literature searches, statistical analyses, and manuscript writing. Hua Gao, Xuequan Chen, Hui Yuan, and Yang Zhang were responsible for clinical‐scale assessment data collection. Junwei Yan and Qingrong Xia were responsible for healthy controls recruitment. All authors have contributed to and have approved the final manuscript.

## Data Availability

The data are not publicly available due to privacy or ethical restrictions.
